# In Skeletal Muscle Fibers, Protein Kinase Subunit CSNK2A1/CK2α Is Required for Proper Muscle Homeostasis and Structure and Function of Neuromuscular Junctions

**DOI:** 10.3390/cells11243962

**Published:** 2022-12-07

**Authors:** Mira Merholz, Yongzhi Jian, Johannes Wimberg, Lea Gessler, Said Hashemolhosseini

**Affiliations:** 1 Institute of Biochemistry, Medical Faculty, Friedrich-Alexander-University of Erlangen-Nürnberg, 91054 Erlangen, Germany; 2 Muscle Research Center, Friedrich-Alexander-University of Erlangen-Nürnberg, 91054 Erlangen, Germany

**Keywords:** CSNK2A1, CSNK2A2, CSNK2B, protein kinase CK2, skeletal muscle, myogenesis, neuromuscular junction

## Abstract

CSNK2 tetrameric holoenzyme is composed of two subunits with catalytic activity (CSNK2A1 and/or CSNK2A2) and two regulatory subunits (CSNK2B) and is involved in skeletal muscle homeostasis. Up-to-date, constitutive Csnk2a2 knockout mice demonstrated mild regenerative impairments in skeletal muscles, while conditional Csnk2b mice were linked to muscle weakness, impaired neuromuscular transmission, and metabolic and autophagic compromises. Here, for the first time, skeletal muscle-specific conditional Csnk2a1 mice were generated and characterized. The ablation of Csnk2a1 expression was ensured using a human skeletal actin-driven Cre reporter. In comparison with control mice, first, conditional knockout of CSNK2A1 resulted in age-dependent reduced grip strength. Muscle weakness was accompanied by impaired neuromuscular transmission. Second, the protein amount of other CSNK2 subunits was aberrantly changed. Third, the number of central nuclei in muscle fibers indicative of regeneration increased. Fourth, oxidative metabolism was impaired, reflected by an increase in cytochrome oxidase and accumulation of mitochondrial enzyme activity underneath the sarcolemma. Fifth, autophagic processes were stimulated. Sixth, NMJs were fragmented and accompanied by increased synaptic gene expression levels. Altogether, knockout of Csnk2a1 or Csnk2b results in diverse impairments of skeletal muscle biology.

## 1. Introduction

The protein kinase CSNK2 (formerly CK2 or Casein kinase 2) is a tetramer composed of 2 catalytically active α- (CSNK2A1 or CK2α) and 2 β-subunits (CSNK2B or CK2β) and is important for cell proliferation, differentiation, and survival [1,2]. The catalytic α-subunits appear in cells with two different subtypes, either as α1- or α - or as α2- or α’- (CSNK2A2 or CK2α’). These two different catalytic subunits are encoded by different genes, either Csnk2a1 or Csnk2a2, respectively. The two β-subunits, encoded by Csnk2b, contribute to substrate specificity, implying the catalytic subunits are constitutively active in their absence. CSNK2 holoenzyme phosphorylates serine or threonine amino acid residues embedded in the consensus sequence x-S/T-x-x-E/D/pS/pY-x [3,4,5]. Previous analysis shows a more dynamic spatiotemporal organization of the individual CSNK2 subunits in living cells integrating into different multi-molecular assemblies [6]. Importantly, stoichiometric expression of the CSNK2 subunits ensures their physiological function, as an excessive expression of catalytic activity containing CSNK2A1/2 subunits is linked to some malignant tumors such as human squamous cell carcinomas, adenocarcinomas, and other miscellaneous tumors [7,8].

The role of CSNK2 in skeletal muscles has not been studied very extensively until now, but the current state of knowledge was recently reviewed [2]. Development of the muscle cells, defined as myogenesis, is a process that can be divided into several distinct phases, such as activation, proliferation, and differentiation of muscle stem cells up to the formation of mature multi-nucleated syncytia, called muscle fibers. Motor nerve ends couple to single muscle fibers and leads to the generation of a neuromuscular synapse, the so-called neuromuscular junction (NMJ) [2]. At the NMJ, several signaling pathways are responsible for ensuring the clustering of nicotinic acetylcholine receptors (AChRs, CHRN) at the postsynaptic apparatus [9]. A neural isoform of a large heparansulfate proteoglycan, called AGRIN, is released by the nerve ending and is involved in both the stabilization of clusters of existing acetylcholine receptors and the stimulation of synaptic gene expression. To this end, AGRIN interacts with its receptor, low-density lipoprotein receptor-related protein 4 (LRP4), and thereby activates the co-receptor MuSK, a muscle-specific receptor tyrosine kinase. The clustering of AChRs serves as a hallmark for the presence of the postsynaptic apparatus within the endplate zone, the central part of each muscle fiber [9]. Activation of the MuSK-LRP4 complex is also involved in the regulation of the prepatterning step, before muscle innervation, during which AChRs begin to aggregate in a central synaptic region of the muscle [10,11,12].

A number of in vitro studies using immortalized cultured C2C12 muscle cells were performed to better understand the role of CSNK2 in skeletal muscle biology [13,14,15,16,17]. Knockout of individual CSNK2 subunits in those cultured muscle cells enabled the performing of proteome and phosphoproteome analysis. Overall, the phosphoproteome analysis of these knockout C2C12 cells suggests that CSNK2 pleiotropy is less pronounced than expected and supports the idea that the phosphoproteome produced by this kinase is flexible and not predetermined and rigid [15].

However, obtaining relevant in vivo data demands the use of animal models. Fortunately, data about the characterization of the knockout mice for CSNK2A2 and CSNKB were reported. The investigation of constitutive Csnk2a2 knockout mice did not reveal any apparent skeletal muscle phenotype, but there was impairment and involvement of CSNK2A2 in muscle regeneration detected [18]. Characterization of the first conditional muscle-specific deletion of Csnk2b in mice helped to understand its role in muscle fibers and at NMJs [19,20,21]. In the absence of CSNK2B in skeletal muscle fibers, conditional Csnk2b knockout mice developed an age-dependent decrease in grip strength accompanied by NMJ fragmentation and impairments of neuromuscular transmission [21,22]. A number of NMJ-linked proteins were identified, all interacting with CSNK2 subunits and partly being phosphorylated by CSNK2 [20,21]. Regarding the role of CSNK2 in adult skeletal muscles, early studies linked CSNK2 activity to metabolism [23]. Later, the conditional CSNK2B mice were used to gain more understanding of the role of CSNK2B in muscle physiology [19,22,24]. A reduction in oxidative enzymatic activity was determined for conditional Csnk2b-deficient muscle fibers in comparison with controls [19,22,24]. Further, analysis of human muscle biopsies points to an eventual involvement of CSNK2 in mitochondrial myopathies [25]. By looking for a mechanism, it turned out that CSNK2B mediated phosphorylation of TOMM22, the central component of the TOMM (translocase of outer mitochondrial membrane) receptor complex, changed its binding affinity for mitochondrial precursor proteins [22] and is a critical switch for mitophagy with physiological implications on metabolism, muscle integrity, and behavior [2].

Here, for the first time, skeletal muscle-specific conditional Csnk2a1 mice were generated and characterized to understand the role of CSNK2A1 in adult skeletal muscle fibers and at NMJs. Conditional muscle-specific knockout of Csnk2a1 resulted in age-dependent muscle weakness and impaired neuromuscular transmission, an aberrant change in protein amount of other CSNK2 subunits, stimulated autophagy processes and fiber regeneration, modulated fiber type distribution, and NMJ fragmentation accompanied by increased synaptic gene expression levels.

## 2. Materials and Methods

### 2.1. Mouse Procedures and Genotyping

Mouse experiments were performed in accordance with animal welfare laws and approved by the responsible local committees (animal protection officer, Sachgebiet Tierschutzangelegenheiten, FAU Erlangen-Nürnberg, AZ: I/39/EE006 and TS-07/11) and government bodies (Regierung von Unterfranken). Floxed mice were kindly provided by Dr. Marc Flajolet [26]. Cre reporter mice were described before [21,27]. Mice were housed in cages that were maintained in a room with a temperature of 22 ± 1 °C and relative humidity of 50–60% on a 12-h light/dark cycle. Water and food were provided ad libitum. Mouse mating and genotyping were performed as previously described [28]. Mice were genotyped using PCR analysis of ear biopsy DNA [21]. The muscle force of the mice was measured with all four limbs using a Grip Strength Test Meter (Bioseb, Chaville, France) [19] and an upside-down grid test [19].

### 2.2. RNA Extraction, Reverse Transcription, PCR

Total RNA was extracted from the hind limb muscle of mice with TRIzol reagent (Thermo Fisher Scientific, Schwerte, Germany, 15596026) [21] and reverse transcribed with M-MuLV Reverse Transcriptase (New England Biolabs, Frankfurt am Main, Germandy, M0253) according to the manufacturer’s instructions. cDNAs were used with mouse-specific primers (Supplementary Table S1) for quantitative PCR reactions using the PowerUp SYBR Green Master Mix (Thermo Fisher Scientific, A25743) and the C1000 Thermal Cycler with the CFX96 Real-Time PCR Detection System (Bio-Rad Laboratories GmbH, Feldkirchen, Germany) according to the manufacturer’s instructions. After the PCR run, the sizes of amplified DNA products were verified using agarose gel electrophoresis. Ct values of the genes of interest were normalized to Ct values of the internal control (Rpl8 gene) (normalized expression = 2^−ΔCT^) [29,30].

### 2.3. Protein Lysates, SDS-PAGE, Western Blot

For the preparation of muscle tissue extract, mouse gastrocnemius and tibialis anterior muscles were dissected, frozen in liquid nitrogen, mashed, and homogenized in ice-cold lysis buffer (10 mM Hepes at pH 7.9, 0.2 mM EDTA, 2 mM DTT, 1% Nonidet P-40, 2 μg/μL Leupeptin and Aprotinin) for 10 min. Protein lysates were diluted with Laemmli buffer, boiled at 95 °C for 5 min, and separated by sodium dodecyl sulfate (SDS) polyacrylamide gel electrophoresis with the Biometra Minigel Twin system. Separated proteins were blotted onto a nitrocellulose membrane (Sigma Aldrich, Taufkirchen, Germany, Protran BA 85), blocked in 5% BSA or 5% non-fat dry milk in TBS with 0.1% Tween20, slowly shaking for 1 h at room temperature. After blocking, the membranes were incubated with primary antibodies at 1:3000 dilution, slowly shaking overnight at 4 °C: CSNK2A1 (Thermo Fisher Scientific, Schwerte, Germany), CSNK2A2, and CSNK2B (generated in the lab of Olaf Issinger, Odense, Denmark), AKT (Cell Signaling, Leiden, The Netherlands, 9272), p-AKT S129 (Sigma Aldrich Chemie, Taufkirchen, Germany), p-AKT S473 (Cell Signaling Technology, Leiden, The Netherlands, 9271), SQSTM1 (Abcam, Berlin, Germany, ab56416), OPTN (Proteintech Germany, Planegg-Martinsried, Germany, 10837-1-AP), MAP1LC3B (Cell Signaling Technology, Leiden, The Netherlands, 2775), GAPDH (Santa Cruz Biotechnology Inc., Heidelberg, Germany, sc-101199). Corresponding HRP-linked secondary antibodies against rabbit (Cell Signaling Technology, Leiden, The Netherlands, 7074) at a 1:3000 dilution were bound for 2 h at room temperature. Protein bands were detected with a chemiluminescence reagent solution, and protein bands were exposed on RXSuper X-Ray films (FUJIFILM Europe GmbH, Ratingen, Germany). The chemiluminescence reagent consisted of 3 ml of 0.25 mg/mL Luminol (Sigma Aldrich, A-4685) in 0.1 M Tris pH 8.6 solution and 40 μL of 1.1 mg/mL Para-hydroxy-cumarinic acid (Sigma Aldrich, C-9008) in DMSO, mixed with 1.2 μL of 30% H_2_O_2_. Western blot results were quantified by densitometric analysis using Fiji software (Vers. 1.53t, 2022) [31]. Films were scanned with an Epson Expression 1600 Pro Scanner (Epson Deutschland GmbH, Meerbusch, Germany) at 800 dpi. After background subtraction, protein bands of interest were labeled and measured. For quantification, the protein band intensity was normalized to the intensity of the GAPDH protein band of the respective sample.

### 2.4. Histochemical Stainings, Immunofluorescence Staining, Fluorescence Microscopy, Quantitative 3D Morphometrical Imaging

Histochemical and immunofluorescence analysis was essentially performed as described [22]; gastrocnemius and soleus muscles were quick-frozen in prechilled isopentane. Cryotome dissected muscles were either used for histochemical or for immunofluorescence stainings. Sections were embedded in DPX or mowiol. Hematoxylin and eosin (H&E) staining: sections were incubated for 15 min in Mayer hemalum solution (Merck, Darmstadt, Germany, 109249), washed 10 min in tap water, dipped 6 times in a solution containing 96% ethanol and 4% HCl, 10 min in tap water, 1 min in 70% ethanol, 2 min in Eosin (Merck, 115935), 1 min in 100% ethanol. Cytochrome oxidase (COX): Sections were incubated for 60 min at 37 °C in a solution containing 50 mM phosphate buffer, pH 7.4, 3,3-di-aminobenzidinetetrahydrochloride (DAB; Sigma Aldrich, D8001), catalase (20 mg/mL; Sigma Aldrich, S41168), sucrose, and CYCS/cytochrome c (Sigma-Aldrich, C2037). Afterward, they were washed in H_2_O and embedded. For immunofluorescence stainings, muscles were fixed in 2% PFA (paraformaldehyde), permeabilized for 15 min in 0.1% Triton X-100 and 100 mM Glycin, and blocked with MOM for 1 h. The following antibodies were used for staining: anti-myosin heavy chain type I (Sigma-Aldrich, M8421, 1:3.000) and anti-myosin heavy chain type II (Sigma-Aldrich, M4276, 1:3000). Secondary antibodies conjugated to Alexa Fluor 546 (A-21123, Invitrogen; 1:1000) were used for detection [22]. Stainings were documented using a Zeiss Axio Examiner Z1 microscope (Carl Zeiss MicroImaging, Göttingen, Germany) equipped with an AxioCam MRm camera (Carl Zeiss MicroImaging) and ZEISS AxioVision Release 4.8 (Carl Zeiss MicroImaging) [19].

For quantitative 3D morphometrical imaging, mouse soleus muscle was dissected and fixed in 2% PFA for 2 h at 4 °C. For detection of AChRs, muscle bundles containing 5–10 fibers were prepared and stained with rhodamine-α-bungarotoxin (BTX) (1:2.500, Invitrogen) for 1 h at room temperature. Stained bundles were washed 3 times for 5 min in PBS, pre-last washing step with PBS and DAPI (Dilution 1:10,000), and embedded in mowiol. Then, 3D images of NMJs were taken with a 63× oil objective (Zeiss Examiner E1, Carl Zeiss MicroImaging) at 55 ms exposure time. Images were deconvoluted and analyzed using different modules in AxioVision software (ZEISS AxioVision Release 4.9, Carl Zeiss MicroImaging). The following parameters were determined for each NMJ: volume, surface, grey sum, grey mean, number of fragments, and number of synaptic nuclei. For each genotype, more than 50 NMJs were analyzed [19].

### 2.5. Nerve Muscle Preparation and Electrophysiological Recordings

Electrophysiological recordings were performed as already reported [19]. Diaphragm–phrenic nerve preparations were maintained ex vivo in Liley’s solution gassed with 95% O_2_ and 5% CO_2_ at room temperature [32]. The recording chamber had a volume of approximately 1 mL and was perfused at a rate of 1 mL/min. The nerve was drawn up into a suction electrode for stimulation with pulses of 0.1 ms duration (4). The preparation was placed on the stage of a Zeiss Axio Examiner Z1 microscope (Carl Zeiss MicroImaging) fitted with incident light fluorescence illumination with filters for 547 nm/red (Zeiss filter set 20) fluorescing fluorophore (Carl Zeiss MicroImaging). The compound muscle action potential (CMAP) was recorded using a micropipette with a tip diameter of approximately 10 μm filled with bathing solution. The electrode was positioned so that the latency of the major negative peak was minimized. The electrode was then positioned 100 μm above the surface of the muscle, and CMAP was recorded. Trains of repetitive nerve stimulations (5 Hz) were performed at 2 min intervals, and the ratio of CMAP amplitudes (mean (20th–25th)/2nd) was calculated [19,33]. To block muscle action potentials so that EPPs (endplate potentials) and EPCs (endplate currents) could be recorded at 1 Hz for 40 s [34,35], μ-conotoxin GIIIB (μ-CTX, 2 μM; Peptide Institute) was added to Lilly’s solution. EPPs were recorded at 5 Hz for 5 s and at 20 Hz for 10 s. Decrements in EPPs were calculated employing the mean of the first and the last five recordings. Concurrently, clustered AChRs at NMJs were labeled by adding 0.5 × 10^−8^ M of BTX (Life Technologies, Darmstadt, Germany) to the same Lilly solution. In some experiments, the effect of the toxin wore off after 1–2 h, and contractions resumed in response to nerve stimulation. These preparations were then exposed a second time to the toxin. Two intracellular electrodes (resistance 10–15 MΩ) were inserted within 50 μm of the NMJs under visual inspection [36]. Current was passed through one electrode to maintain the membrane potential within 2 mV of −75 mV, while voltage transients were recorded with the other. Signals were amplified by an Axoclamp 900 A and digitized at 40 kHz by a Digidata 1440 A under the control of pCLAMP 10 (Molecular Devices, Sunny Vale, CA, USA). Voltage records were filtered at 3 kHz, and current records at 1 kHz (8-pole Bessel filter). Current transients were recorded using the two-electrode voltage-clamp facility of the Axoclamp 900 A. Clamp gains were usually 300–1000, reducing the voltage transients to< 3% of their unclamped amplitudes. At most NMJs, 50–100 spontaneous quantal events were recorded during a period of 1 min. Records were analyzed using pCLAMP 10. Spontaneous events were extracted using the “template search” facility and edited by eye to remove obvious artifacts. Events recorded from each NMJ were averaged, and the amplitude and frequency were determined [19].

### 2.6. Statistical Analysis

Statistical analysis was performed in GraphPad Prism 9 (GraphPad Software, San Diego, USA) as indicated. Data are presented as mean values, and the error bars indicate S.D. The number of biological replicates per experimental variable (*n*) is usually *n* > 5 or as indicated in the figure legends. For all data with mice, a minimum of 3 mice were studied. The significance is calculated by unpaired 2-tailed Student’s *t*-test, or as indicated by the figure legends, and provided as real *p*-values that are believed to be categorized for different significance levels, **** *p* < 0.0001, *** *p* < 0.001, ** *p* < 0.01, or * *p* < 0.05.

## 3. Results

### 3.1. Age-Dependent Impairment of Muscle Grip Strength in Conditional Skeletal Muscle-Specific Csnk2a1 Knockout Mice

**Figure 1:**
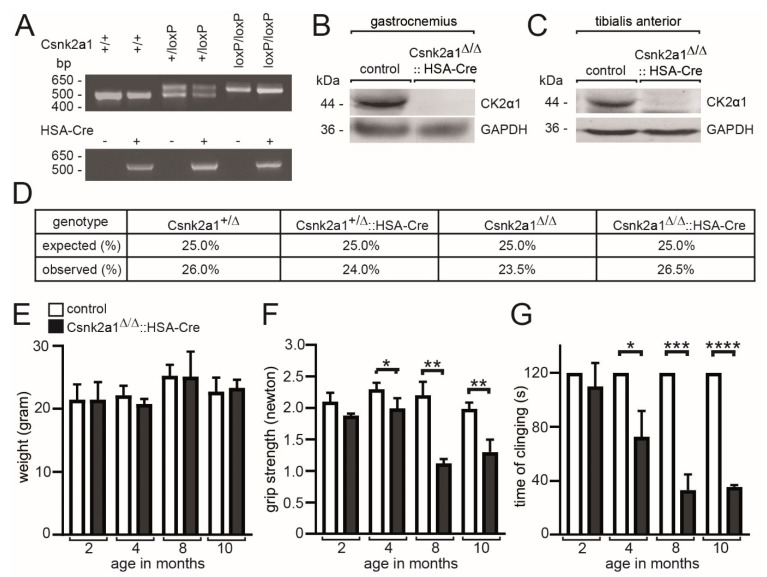
Phenotype of conditional Csnk2a1 knockout mice. (**A**) Agarose gel image shows amplified DNA fragments detected by PCR genotyping of the heterozygous and homozygous floxed alleles of Csnk2a1. Representative images of Western blot membranes reflect the amount of CSNK2A1 and GAPDH protein in gastrocnemius (**B**) or tibialis anterior (**C**) muscle lysates of conditional Csnk2a1 knockout and control mice. (**D**) Expected and observed distribution frequencies of breedings with conditional Csnk2a1 knockout mice are presented. (**E**) Graph presents the body weight of control and conditional Csnk2a1 knockout mice at indicated ages. (**F**) Muscle force is presented by a graph summarizing grip strength for control and conditional Csnk2a1 knockout mice. Note, in comparison with controls, the grip strength of conditional Csnk2a1 knockout mice is gradually reduced starting by the age of 4 months. (**G**) Graph presents muscle force as reflected by the clinging time of mice upside-down on a grid. All labeling of graph bars represents the genotype of mice as indicated in (**E**). Data are represented as mean ± S.D.; *n* ≥ 3 mice per genotype. **** *p* < 0.0001, *** *p* < 0.001, ** *p* < 0.01, * *p* < 0.05.

Previously, conditional Csnk2b skeletal muscle knockout mice were generated by breeding floxed mice with human skeletal actin (HSA) Cre reporter mice [37] and were shown to have less muscle grip strength [21]. We started generating conditional Csnk2a1 knockout mice using the same HSA-Cre reporter mice. Genotyping PCRs using genomic DNA from tissue biopsies of mice confirmed Csnk2a1 alleles being floxed in hetero- or homozygous fashion (Figure 1A). Genome editing of the Csnk2a1 locus was paralleled by the ablation of CSNK2A1 protein analyzing two different skeletal muscles, gastrocnemius and tibialis anterior (Figure 1B,C). Calculation of offspring numbers demonstrated mendelian distribution frequencies and neither lethality nor haploinsufficiency at this level (Figure 1D). Characterizing conditional Csnk2a1 knockout mice in comparison with control mice, we did not detect any change in body weight, regardless of age (Figure 1E). Interestingly, grip strength, measured by a Newton meter and by measuring the time mice were able to hang upside-down on a grid, gradually decreased depending on their age in conditional Csnk2a1 knockout mice (Figure 1F,G). These data demonstrate CSNK2A1 being important for muscle grip strength.

### 3.2. Modulated Transcript and Protein Amounts of Csnk2a2 and Csnk2b, and CSNK2 Target Protein AKT1, in the Absence of Csnk2a1 in Hind Limb Muscles of Conditional Knockout Mice

**Figure 2:**
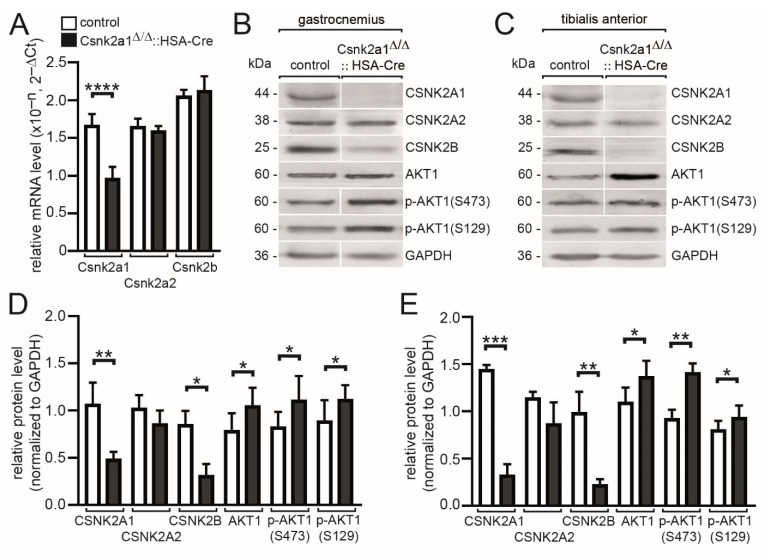
mRNA and protein amounts of other subunits of CSNK2 and a typical target of CSNK2, AKT1, both in conditional Csnk2a1 knockout mice. (**A**) Graph summarizes transcript amounts as detected by qPCR experiment using extracted RNA from tibialis anterior muscle of conditional Csnk2a1 knockout and control mice. (**B**,**C**) Images of typical Western blot membranes using muscle lysates from gastrocnemius or tibialis anterior muscles. While CSNK2A1 protein is absent in conditional Csnk2a1 knockout muscle lysates as expected, the total amount of CSNK2B is very low. CSNK2A2 protein amount appears only slightly reduced. Surprisingly, the amount of AKT1 is higher in conditional Csnk2a1 knockout muscle lysates in comparison with controls, and even the amount of phospho-AKT(S129) protein, a typical CSNK2 target, is significantly increased. (**D**,**E**) Graphs present densitometrical quantifications of protein amounts as detected by Western blot images, as shown in (**B**,**C**). All labeling of graph bars represents the genotype of mice as indicated in (**A**). Data are represented as mean ± S.D.; *n* ≥ 5 mice per genotype. **** *p* < 0.0001, *** *p* < 0.001, ** *p* < 0.01, * *p* < 0.05.

Previous reports suggest a different response of individual CSNK2 subunit protein amount after the knockout of one of the CSNK2 subunits [14,17,22]. First, we performed quantitative PCR studies and confirmed significantly lower Csnk2a1 mRNA amounts in conditional Csnk2a1 knockout mice (Figure 2A). The transcript amount for Csnk2a2 and Csnk2b was not affected (Figure 2A). In parallel, looking for protein amounts after normalization, a significantly lower CSNK2B level was detected in gastrocnemius and tibialis anterior muscles (Figure 2B,C). The protein amount of CSNK2A2 was only slightly, but not significantly, reduced in the absence of CSNK2A1 in the same two muscles (Figure 2D,E). The Rho family-alpha serine/threonine-protein kinase AKT1 is believed to be a typical target of CSNK2 and is mainly phosphorylated at serine 129 in several immortalized cultured cell lines [38]. Serine 473 of AKT1 was reported to be not phosphorylated by CSNK2 [38]. Unexpectedly, phospho-AKT1 serine 129 and 473 protein are both upregulated in conditional Csnk2a1 knockout gastrocnemius and tibialis anterior muscles (Figure 2B,C). Total AKT1 protein amount was also significantly upregulated in muscle tissues of conditional Csnk2a1 knockout mice in comparison with controls (Figure 2D,E).

### 3.3. Increased Regeneration, More Oxidative Metabolism, and Modified Fiber Type Distribution and Cross-Sectional Area in Conditional Csnk2a1 Knockout Mice

**Figure 3:**
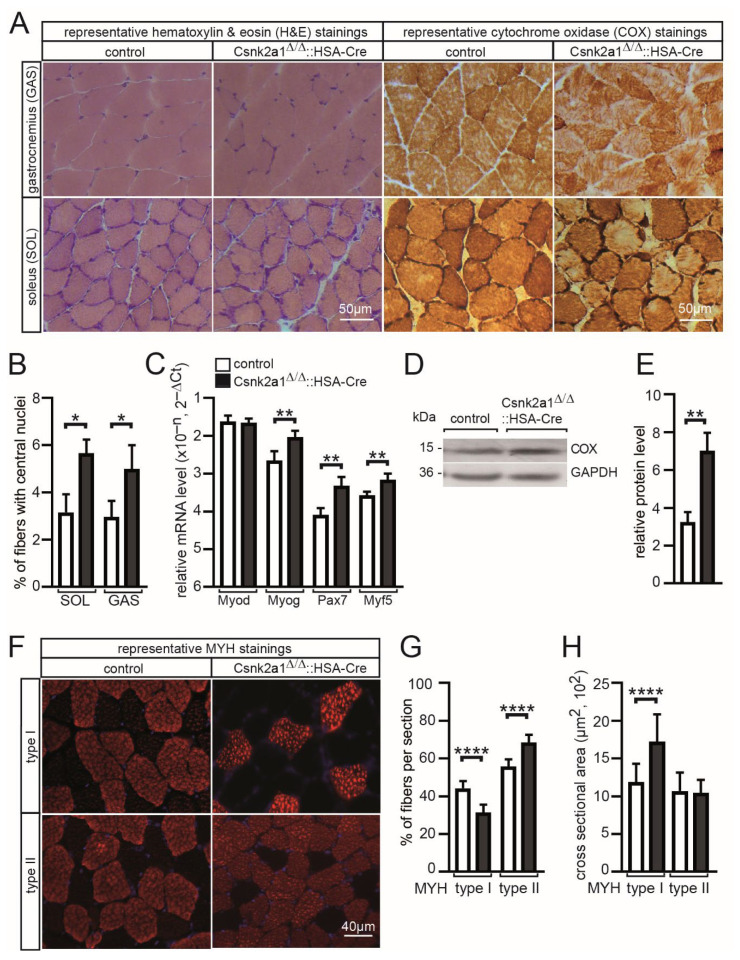
Increased number of central nuclei, cytochrome oxidase, and several myogenic markers are accompanied by changes in muscle fiber distribution and cross-sectional areas in conditional Csnk2a1 knockout mice. (**A**) Hematoxylin/eosin (H&E) and cytochrome oxidase (COX) stains of hind limb muscle cross-sections of control and conditional Csnk2a1 knockout mice. (**B**) Graph presents the number of central nuclei as counted in a representative number of cross-sections of control and conditional Csnk2a1 knockout mice. (**C**) Graph summarizes transcript amounts of myogenic markers Myod1, Myog, Pax7, and Myf5 in tibialis anterior muscle of control and conditional Csnk2a1 knockout mice. (**D**) Image of Western blot ascertains a higher amount of cytochrome oxidase in conditional Csnk2a1 knockout gastrocnemius muscle lysates in comparison with controls. (**E**) Graph presents densitometrical quantification of representative images of Western blots as shown in (**D**). (**F**) Immunofluorescence stainings of soleus muscle cross sections for detection of myosin heavy chain stained fibers. (**G**) Graph summarizes distribution of type I and type II fibers in soleus muscles of conditional Csnk2a1 knockout and control mice. (**H**) Cross-sectional areas (CSA) were measured and are presented against muscle fiber types I and II. All labeling of graph bars represents the genotype of mice as indicated in (**C**). Data are represented as mean ± S.D.; *n* ≥ 5 mice per genotype. **** *p* < 0.0001, ** *p* < 0.01, * *p* < 0.05.

In conditional CSNK2B muscle-specific knockout mice, there is reduced oxidative metabolism activity and accelerated mitophagy [22,24]. To determine whether similar changes happen in the absence of CSNK2A1 in skeletal muscle, gastrocnemius and soleus were cross-sectioned, and different histochemical stainings were performed. Using hematoxylin and eosin staining, no obvious changes in histology were visible (Figure 3A), but after counting the number of central nuclei, indicating regenerated muscle fibers, a significantly higher number of central nuclei was detected in gastrocnemius and soleus muscle of conditional CSNK2A1 knockout mice in comparison with controls (Figure 3B). We wondered whether this indicated regeneration is associated with a higher number of muscle satellite cells. Indeed, transcript amounts of myogenic markers, such as Pax7, Myog, and Myf5, were upregulated in conditional Csnk2a1 knockout mice (Figure 3C). After employing cytochrome oxidase (COX) staining, several changes were evident (Figure 3A). Subcellular COX staining was different in conditional CSNK2A1 knockout mice; it appears the subsarcolemmal staining pattern is strongly increased (Figure 3A), and this correlated with a significantly higher amount of cytochrome oxidase detected by Western blot using muscle lysates of conditional Csnk2a1 knockout and control mice (Figure 3D,E). Next, we wanted to know whether conditional Csnk2a1 knockout mice fiber type distribution or fiber type-dependent cross-sectional areas (CSA) were compromised. Interestingly, soleus fibers contained a significantly lower number of slow type I fibers but an increased number of fast type II in conditional Csnk2a1 knockout mice in comparison with control mice (Figure 3F,G). Moreover, type I fiber CSA was significantly increased in conditional Csnk2a1 knockout mice (Figure 3F,H). In sum, these data indicate a role of CSNK2A1 in apoptotic events resulting in skeletal muscle fiber regeneration because HSA-Cre mediated Csnk2a1 knockout occurs in myotubes during development, not in muscle satellite cells. CSNK2A1 also appears involved in oxidative metabolism and fiber-type cross-sectional area and distribution.

### 3.4. Muscle Weakness of Conditional Csnk2a1 Mice Is Linked to NMJ Fragmentation and Increased Synaptic Gene Expression

**Figure 4:**
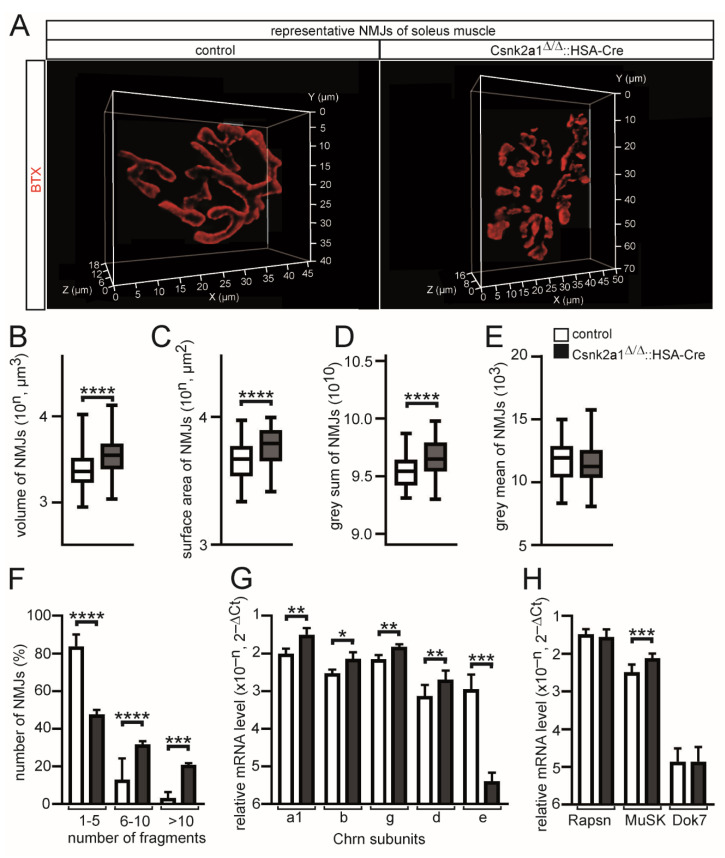
NMJs of conditional Csnk2a1 knockout mice are heavily fragmented and accompanied by modulated synaptic expression of all Chrn transcripts and receptor tyrosine kinase MuSK. (**A**) Typical 3D images from an individual NMJ of a control and a conditional Csnk2a1 knockout soleus muscle fiber are presented. (**B**–**E**) Graphs summarize quantitative 3D imaging parameters of NMJs of soleus muscle fibers of conditional Csnk2a1 knockout mice in comparison with controls. Note, NMJ volume, surface area, and grey sum are increased in conditional Csnk2a1 knockout mice. (**F**) NMJ fragmentation grade is presented by a graph subgrouping three different ranges of NMJ fragment numbers. (**G**,**H**) Total RNA was extracted, 1st cDNA generated, and qPCR experiment performed. Graphs summarize changes in transcription levels of myogenic and synaptic genes as indicated. Note, Chrne is significantly downregulated, while all other Chrn genes and Musk are upregulated in conditional Csnk2a1 knockout hindlimb muscles. All labeling of graph bars represents the genotype of mice as indicated in (**E**). Data are represented as mean ± S.D.; *n* ≥ 5 mice per genotype and analysis of >150 individual NMJs per muscle. **** *p* < 0.0001, *** *p* < 0.001, ** *p* < 0.01, * *p* < 0.05.

In order to find out whether conditional Csnk2a1 knockout mice are accompanied by compromised neuromuscular structure and function, typical pretzel-shaped NMJs of the soleus muscle of control and conditional Csnk2a1 knockout mice were dissected and stained using BTX. During aging or due to pathologies, pretzel-shaped NMJs might be fragmented [9]. Of note, NMJs of conditional Csnk2a1 knockout soleus muscles are significantly fragmented (Figure 4A,F). After quantitative 3D imaging of NMJs of soleus fibers, volume, surface area, and grey sum of fluorescence intensity were significantly increased in conditional Csnk2a1 knockout mice in comparison with controls (Figure 4B–D). Grey mean values of NMJs were not changed between conditional Csnk2a1 knockout mice and control NMJs (Figure 4E). To find out whether these structural changes are linked to different amounts of synaptic mRNA, qPCR was employed to compare mRNA levels of conditional Csnk2a1 knockout mice and control. Acetylcholine receptor mRNA levels were detected being increased for Chrna1, Chrnb, Chrng, and Chnrd, but Chrne was much lower in conditional Csnk2a1 knockout mice in comparison with controls (Figure 4G). Moreover, synaptic mRNA levels were not changed for Rapsn and Dok7 but for the muscle-specific receptor tyrosine kinase, Musk (Figure 4H). These data demonstrate a role for CSNK2A1 regarding the structural integrity of the postsynaptic part of NMJs.

### 3.5. Electrophysiological Recordings of Csnk2a1 Diaphragms Reveal Impaired Amplitudes of Miniature Endplate Potentials, Endplate Potentials, and Decreased Decrements of Endplate Potentials and Compound Muscle Action Potential Amplitudes

**Figure 5:**
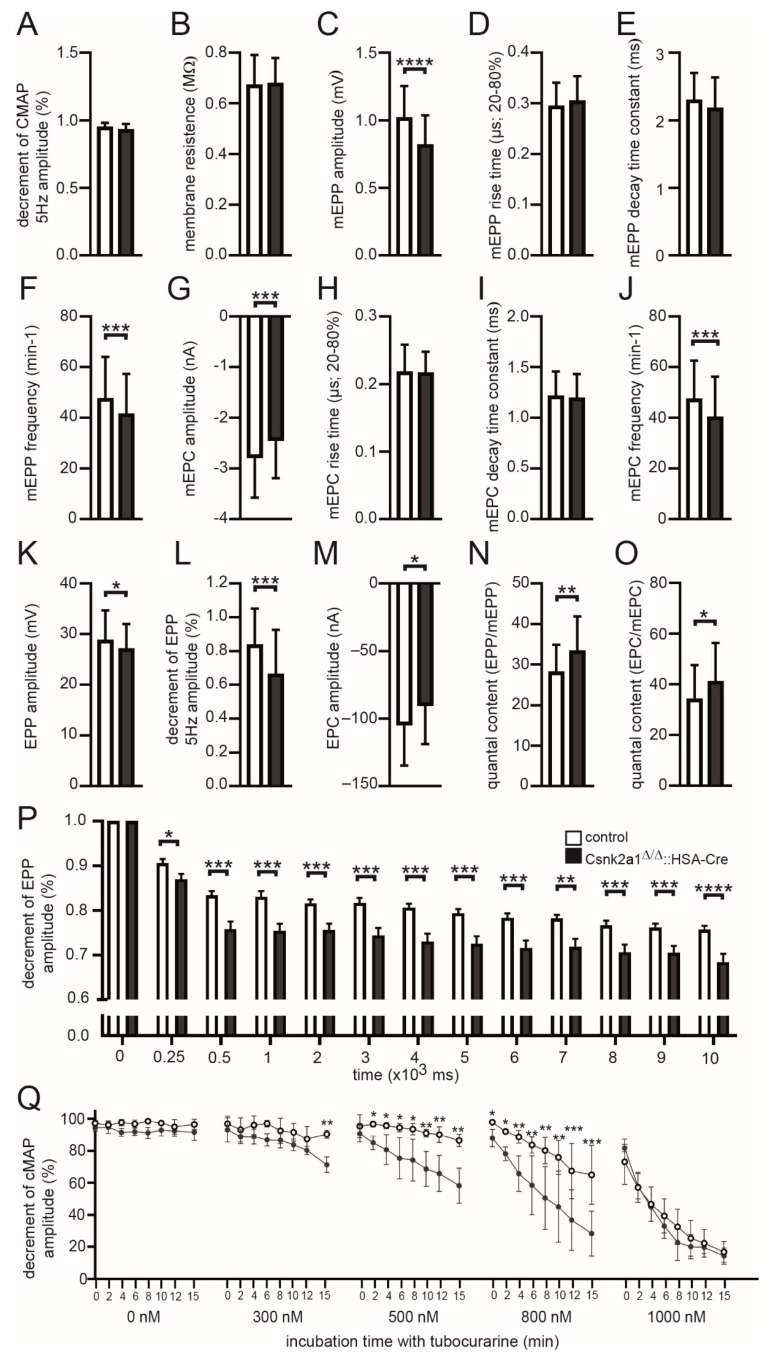
Characterization of neural transmission recording of conditional Csnk2a1 knockout mice. Diaphragms of adult mice of indicated genotypes were employed for electrophysiological recordings. Graphs present the analysis of the following parameters, (**A**) decrement of compound muscle action potential (CMAP) amplitude at 5 Hz, (**B**) membrane resistance, (**C**) miniature endplate potential (mEPP) amplitude, (**D**) mEPP rise time, (**E**) mEPP decay time constant, (**F**) mEPP frequency, (**G**) miniature endplate current (mEPC) amplitude, (**H**) mEPC rise time, (**I**) mEPC decay time constant, (**J**) mEPC frequency, (**K**) endplate potential (EPP) amplitude at 1 Hz, (**L**) decrement of EPP amplitude at 5 Hz, (**M**) endplate current (EPC) amplitude, (**N**,**O**) quantal content (EPP/mEPP or EPC/mEPC). Note, quantal content is increased in conditional Csnk2a1 knockout mice because of impaired amplitudes of end plate and miniature end plate potentials/currents. (**P**) Run-down experiments (decrement of EPP amplitudes) demonstrate a significant decrease in conditional Csnk2a1 knockout mice in comparison with control mice. (**Q**) CMAP measurements in control and conditional Csnk2a1 knockout diaphragms under untreated conditions and in the presence of increasing concentrations of d-tubocurarine. Conditional Csnk2a1 knockout mice showed a significantly higher decrease in CMAP, which is already displayed at 300 nM d-tubocurarine, thus unmasking a reduced safety factor in the absence of Csnk2a1. Data are represented as mean ± S.D.; *n* ≥ 5 mice per genotype and analysis of >20 individual NMJs per muscle. All labeling of graph bars represents the genotype of mice as indicated in (**P**). **** *p* < 0.0001, *** *p* < 0.001, ** *p* < 0.01, * *p* < 0.05.

Previously, decreased miniature endplate current amplitudes were recorded by comparing diaphragm muscle fibers of conditional CSNK2B knockout mice with control mice [21,22]. Here, we addressed whether neuromuscular transmission is impaired in fragmented NMJs of conditional CSNK2A1 knockout mice. We recorded extra- and intra-cellular potentials and currents in muscles of conditional Csnk2a1 knockout mice in comparison with controls to analyze the physiology of neuromuscular transmission at their NMJs (Figure 5). By recording CMAPs, compound muscle action potentials that are triggered by consecutive nerve stimuli, we did not detect a difference between control and conditional Csnk2a1 knockout mice with CMAP amplitude (Figure 5A). Membrane resistance values were comparable between conditional Csnk2a1 knockout and control diaphragms, arguing for non-affected membrane integrity (Figure 5B). Recording of miniature endplate potentials and currents (mEPP, mEPC) revealed a small but significant decrease in their frequency (Figure 5F,J). Moreover, mEPP and mEPC amplitudes slightly decreased in conditional Csnk2a1 knockout mice, arguing for affected local depolarizations around endplates in response to spontaneous acetylcholine release (Figure 5C,G). However, mEPP and mEPC rise time and decay time constants were not changed in conditional Csnk2a1 knockout mice in comparison to controls (Figure 5D,E,H,I). EPP and EPC amplitudes, local responses at NMJs to nerve stimulation, were decreased in conditional CSNK2A1 knockout mice (Figure 5K–M). Quantal content, the mean number of quanta that are released to generate an EPP or EPC, was increased in conditional Csnk2a1 knockout mice in comparison with controls (Figure 5N,O). To measure muscle fatigability in more detail, run-down experiments were performed, as described before [36], and significantly reduced decrement of EPP amplitude recorded in CSNK2A1 diaphragm fibers in comparison to controls (Figure 5P). In agreement, the decrement in CMAP amplitudes was measured after incubation of diaphragms with tubocurare in a dose-dependent fashion (Figure 5Q), as described before [19,39], and were significantly reduced in diaphragms of conditional CSNK2A1 knockout mice. In sum, these results indicate an impaired neuromuscular transmission for conditional CSNK2a1 knockout mice compared to controls, including a significantly higher fatigability.

### 3.6. Mitophagy Appears Upregulated in Conditional Csnk2a1 Diaphragms and Is Accompanied by Increased Amount of General Autophagy Marker SQTM1

**Figure 6:**
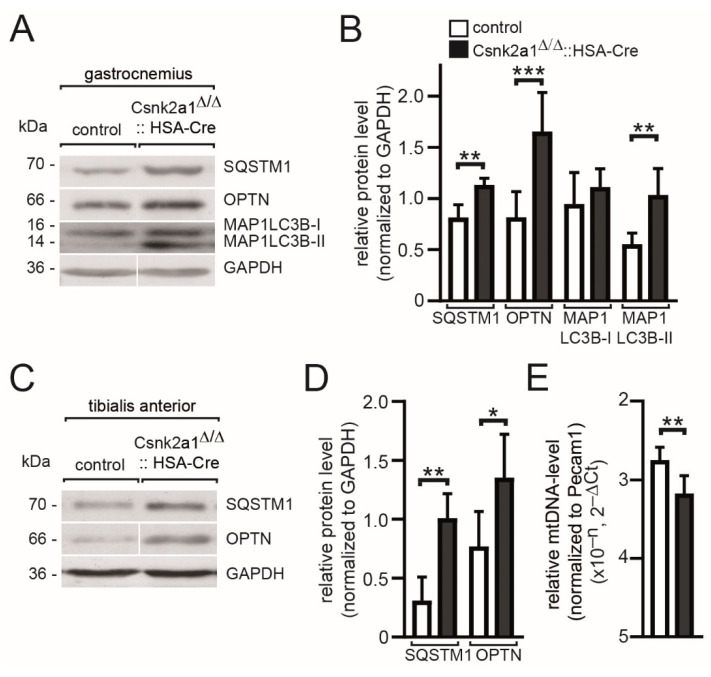
Autophagy is impaired in conditional Csnk2a1 knockout mice. (**A**) Gastrocnemius muscle lysates were used for SDS-PAGE and Western blot; membranes were probed with antibodies labeling proteins implicated in autophagy, namely MAP1LC3B-I/II, SQSTM1, OPTN, and for normalization, GAPDH. (**B**) Graph summarizes quantification of protein bands as seen in (**A**) using Fiji. (**C**) Similar to in (**A**) with muscle lysates of tibialis anterior. (**D**) Graph quantitates protein amounts as shown in (**C**). (**E**) Graph shows the result of qPCR approach to compare the amount of mitochondrial DNA between conditional Csnk2a1 knockout and control tibialis anterior and extensor digitorum longus muscle. Note, the increased amount of MAP1-LC3BII, SQSTM1, and OPTN refers to impaired mitophagy. All labeling of graph bars represents the genotype of mice as indicated in (**B**). Data are represented as mean ± S.D.; *n* ≥ 5 mice per genotype. *** *p* < 0.001, ** *p* < 0.01, * *p* < 0.05.

Mitophagy is impaired in conditional Csnk2b muscle-specific knockout mice due to a lack of CSNK2-dependent phosphorylation of members of the outer membrane mitochondrial import machinery apparently regulating mitochondrial homeostasis [22]. We used muscle lysates of either gastrocnemius or tibialis anterior to analyze whether a similar phenotype is detectable in conditional Csnk2a1 knockout mice (Figure 6). We looked for the number of markers, such as P62/SQSTM1, an autophagic receptor protein that is a well-known substrate of the autophagolysosome system, Optineurin/OPTN, involved in the removal of impaired mitochondria, and LC3/MAP1LC3B/LC3B (microtubule-associated protein 1 light chain 3 beta), which is involved during autophagosome formation and recruited to phagophore membranes [40]. MAP1LC3B exists in 2 forms, the non-lipidated form I and the lipidated form II; the latter is anchored in phagophore and autophagosomal membranes. We analyzed in both muscles an increase of SQSTM1, OPTN, and MAP1LC3B-II in conditional Csnk2a1 knockout muscles in comparison with control muscles (Figure 6A–D). The increased amounts of these markers in conditional Csnk2a1 knockout mice fed ad libitum suggested impaired autophagy eventually due to a block of the autophagic flux. This hypothesis is further evidenced by decreased amounts of mitochondrial genomic DNA, quantified by qPCR, in conditional Csnk2a1 knockout mice in comparison with controls (Figure 6E).

## 4. Discussion

Protein kinase CSNK2 plays a fundamental role in the biology of tissues and cells, which is evident from the phenotype of knockout mice. The tetrameric holoenzyme is composed of catalytic activity containing CSNK2A1 or CSNK2A2 subunits and so-called CSNK2B regulatory subunits [2]. Csnk2a1 knockout mice are lethal during early development [41]. Csnk2a2 knockout mice are infertile due to globozoospermia [42]. Csnk2b knockout mice are lethal because the development of the embryos ends at the blastocyst stage [43]. Indeed, only Csnk2a2 knockout mice were viable, but their analysis revealed perturbation of skeletal muscle regeneration, probably due to compromised cell cycle kinetics [18]. Accordingly, CSNK2A2 appears to have a less important role in adult skeletal muscle fibers. Understanding the role of individual CSNK2 subunits in skeletal muscle cells demanded the analysis of conditional muscle-specific knockout mice using corresponding skeletal muscle lineage-specific Cre reporters. A few years ago, the first conditional Csnk2 subunit skeletal muscle-specific knockout mice were generated with the help of HSA-Cre reporter mice [21]. These conditional Csnk2b knockout mice have significantly less grip strength for many reasons. At least two of the causes were reported. First, disturbed mitochondrial protein import and mitophagy were detected as a cause of the oxidative metabolic disorder in the absence of CSNK2B in vivo in adult muscle fibers [22,24,25], and second, CSNK2B interacts with and also phosphorylates several synaptic proteins at NMJs thereby being involved in the formation and/or maintenance of NMJs [19,20,21].

Here, we generated and investigated for the first time skeletal muscle-specific CSNK2A1 knockout mice using the same HSA-Cre reporter, which was previously used for muscle-specific knockout of CSNK2B [21]. Importantly, these conditional Csnk2a1 knockout mice still are able to express CSNK2A2, which is also known to harbor CSNK2-specific catalytic activity [44,45]. Whether CSNK2A2 catalytic activity replaces CSNK2A1 activity of conditional Csnk2a1 knockout mice cannot be answered here, but several pieces of evidence argue against that. In vitro data obtained by using cultured muscle cells always point to significantly less protein amount of CSNK2B subunits through accelerated proteasomal degradation once CSNK2A1 or CSNK2A2 are knocked out [13,14,17]. Moreover, the knockout of CSNK2B resulted in the complete absence of CSNK2A2 [13,14]. In vivo data are partly contradictory, as in the absence of CSNK2B in adult muscle fibers, more or less CSNK2A subunits are detectable in age-dependent fashion [22]. Interestingly, if the absence of CSNK2A1 consequences in proteasomal removal of CSNK2B, then eventually, available CSNK2A2 activity cannot be part of a tetrameric CSNK2B containing holoenzyme. However, the CSNK2A2 protein amount looks only slightly, but not significantly, reduced in the absence of CSNK2A1 (Figure 2B–E). The CSNK2B protein amount was almost gone in conditional CSNK2A1 knockout mice (Figure 2B–E). Some in vitro evidence suggest that CSNK2A1 monomers or CSNK2B dimers might exist independent from CSNK2 holoenzyme and may possess physiological roles [46,47]. In vitro, it was also shown that CSNK2A1 alone is strongly less efficient in phosphorylating its target proteins in the absence of CSNK2B [21,48]. Whether monomeric or dimeric CSNK2A2 is, in vivo, under physiological conditions, able to phosphorylate its targets in the absence of CSNK2B is completely unknown.

In this context, one might wonder about the phosphorylation status of typical CSNK2 target proteins in the absence of CSNK2A1 in adult muscle fibers. Surprisingly, phosphorylation of AKT at serine 129, described as a typical target of CSNK2 [38], is not downregulated in conditional Csnk2a1 knockout mice (Figure 2B,E). Even if one assumes that CSNK2A2 is responsible for the phosphorylation of AKT at serine 129, it is difficult to explain how this happens in the absence of CSNK2B (Figure 2B,E). It would be interesting to know whether AKT phosphorylation status is different in conditional Csnk2b knockout mice.

The phenotype of conditional Csnk2a1 knockout mice is related to muscle weakness and less muscle grip strength (Figure 1F,G). Interestingly, grip strength was much more strongly reduced in conditional Csnk2b knockout mice [21] in comparison with conditional Csnk2a1 mice. Reduction in grip strength is in both conditional knockout mice linked to fragmented NMJs (Figure 4A,F) [21] and impaired neuromuscular transmission (Figure 5) [19,21]. Moreover, fiber type distribution (Figure 3G) and cross-sectional areas (Figure 3H) are also affected in conditional Csnk2a1 mice; similar changes were detected in conditional Csnk2b knockout mice (data not shown). Histological staining using hematoxyline and eosin demonstrated interstitial fibrosis and fibers being split in Csnk2b mutant mice [22]; these kinds of changes were not detected in Csnk2a1 mutant mice (Figure 3A). Similar to conditional Csnk2b knockout mice, conditional Csnk2a1 knockout mice are associated with compromised oxidative metabolism and mitophagy (Figure 3A and Figure 6A–D). Interestingly, oxidative metabolism appears to be differently impaired in conditional Csnk2a1 knockout mice in comparison with conditional Csnk2b knockout mice. While oxidative metabolism appeared to be replaced by glycolytic metabolism and oxidative fibers were less COX stained in conditional Csnk2b knockout mice [22], more COX stained fibers are still visible in soleus muscles of conditional Csnk2a1 knockout mice (Figure 3A). Of note, positive COX staining is different in comparison with Csnk2b mutant mice [22] regarding subcellular localization, pointing to mitochondrial oxidative activity being accumulated underneath the sarcolemma (Figure 3A). It remains to be investigated by high-resolution electron microscopy whether and how mitochondrial morphology and subcellular localization are affected in conditional Csnk2a1 knockout mice, especially in comparison to conditional Csnk2b knockout mice.

Altogether, the phenotype of conditional muscle-specific Csnk2a1 knockout mice is not simply similar to conditional Csnk2b knockout mice. However, how muscle impairments of conditional Csnk2a1 knockout mice fit to unchanged phosphorylation of Csnk2 target AKT at serine 129 requires further research and might indicate that AKT is not a typical Csnk2 target in adult skeletal muscle fibers.

## Data Availability

Not applicable.

## Supplementary Materials

The following supporting information can be downloaded at: https://www.mdpi.com/article/10.3390/cells11243962/s1, Table S1: Tabular presentation of oligonucleotide sequences.
