# Supplementary material for: In Skeletal Muscle Fibers, Protein Kinase Subunit CSNK2A1/CK2α Is Required for Proper Muscle Homeostasis and Structure and Function of Neuromuscular Junctions
Source: Cells. 2022 Dec 7;11(24):3962. doi: 10.3390/cells11243962 (PMC9776919; doi:10.3390/cells11243962)
Supplement: Supplementary file 1 [file cells-11-03962-s001.zip › cells-2035619-supplementary.pdf]

SUPPLEMENTARY DATA

Supplementary Table S1. Tabular presentation of oligonucleotide sequences.

Genomic target	Orientation	Sequence
Rpl8 qPCR	forward	GTTCGTGTACTGCGGCAAGA
	reverse	ACAGGATTCATGGCCACACC
Csnk2a1 qPCR	forward	CAGCAGCAATCACTGGTGAG
	reverse	TGAGGATAGCCAAGGTTCTG
Csnk2a2 qPCR	forward	TGACCAGCTTGTTCGAATTGCC
	reverse	TGTTCTCAGCACAAGGCTGG
Csnk2b qPCR	forward	TCTGTGAGGTGGATGAAGAC
	reverse	TGTGGATGCACCATGAAGAG
Myod1 qPCR	forward	TGGCATGATGGATTACAGCGG
	reverse	GGTCTGGGTTCCCTGTTCTG
Myog qPCR	forward	CAGTACATTGAGCGCCTACA
	reverse	GCCTGACAGACAATCTCAGT
Pax7 qPCR	forward	GCTACCAGTACAGCCAGTATG
	reverse	GTCACTAAGCATGGGTAGATG
Myf5 qPCR	forward	ATCCGCTACATTGAGAGCCT
	reverse	GATGGCTCTGTAGACGTGAT
Chrna1 qPCR	forward	ACGCTGAGCATCTCTGTCTT
	reverse	TTGGACTCCTGGTCTGACTT
Chrnb qPCR	forward	ATAGGTACCCAGCATACCATATCAGAACG
	reverse	ATACTCGAGCTGAACGGATCAAGAACCAC
Chrng qPCR	forward	GGTCAATGTCAGCCTGAAGC
	reverse	GCACATGCATCCGTAACAGC
Chrnd qPCR	forward	ATGAGGAACAAAGGCTGATCCA
	reverse	ACAGTGATGTTCCCGAAGTCGT
Chrne qPCR	forward	ATAGGTACCCAAGTCCGTTCCTGAGCACG
	reverse	ATACTCGAGCAAGTATCTGCAGCAGGCCA
Rapsn qPCR	forward	CCGCTACAGGCACTCTGTCT
	reverse	TCAGTCTCCTCCACGCACTC
Musk qPCR	forward	GCCTTGGTTGAAGAAGTAGC
	reverse	CTTGATCCAGGACACAGATG
Dok7 qPCR	forward	GAATTCGGTTCTCTGCTCAGTCTG
	reverse	CCAAGTCCATGTAGTGCAGCTG
